# Multifunctional Contribution of the Inflated Fruiting Calyx: Implication for Cuticular Barrier Profiles of the Solanaceous Genera *Physalis, Alkekengi*, and *Nicandra*

**DOI:** 10.3389/fpls.2022.888930

**Published:** 2022-07-06

**Authors:** Aline Xavier de Souza, Markus Riederer, Jana Leide

**Affiliations:** Department for Botany II, Julius-von-Sachs-Institute for Biosciences, University of Würzburg, Würzburg, Germany

**Keywords:** *Physalis*, *Alkekengi*, *Nicandra*, inflated fruiting calyx, leaf, fruit, plant cuticle, wax composition

## Abstract

Pivotal barrier properties of the hydrophobic plant cuticle covering aerial plant surfaces depend on its physicochemical composition. Among plant species and organs, compounds of this boundary layer between the plant interior and the environment vary considerably but cuticle-related studies comparing different organs from the same plant species are still scarce. Thus, this study focused on the cuticle profiles of *Physalis peruviana, Physalis ixocarpa, Alkekengi officinarum*, and *Nicandra physalodes* species. Inflated fruiting calyces enveloping fruits make *Physalis, Alkekengi*, and *Nicandra* highly recognizable genera among the Solanoideae subfamily. Although the inflation of fruiting calyces is well discussed in the literature still little is known about their post-floral functionalities. Cuticular composition, surface structure, and barrier function were examined and compared in fully expanded amphistomatous leaves, ripe astomatous fruits, and fully inflated hypostomatous fruiting calyces. Species- and organ-specific abundances of non-glandular and glandular trichomes revealed high structural diversity, covering not only abaxial and adaxial leaf surfaces but also fruiting calyx surfaces, whereas fruits were glabrous. Cuticular waxes, which limit non-stomatal transpiration, ranged from <1 μg cm^−2^ on *P. peruviana* fruiting calyces and *N. physalodes* fruits to 22 μg cm^−2^ on *P. peruviana* fruits. Very-long-chain aliphatic compounds, notably *n*-alkanes, *iso*-, and *anteiso*-branched alkanes, alkanols, alkanoic acids, and alkyl esters, dominated the cuticular wax coverages (≥86%). Diversity of cuticular wax patterns rose from leaves to fruiting calyces and peaked in fruits. The polymeric cutin matrix providing the structural framework for cuticular waxes was determined to range from 81 μg cm^−2^ for *N. physalodes* to 571 μg cm^−2^ for *A. officinarum* fruits. Cuticular transpiration barriers were highly efficient, with water permeabilities being ≤5 × 10^−5^ m s^−1^. Only the cuticular water permeability of *N. physalodes* fruits was 10 × 10^−5^ m s^−1^ leading to their early desiccation and fruits that easily split, whereas *P. peruviana, P. ixocarpa*, and *A. officinarum* bore fleshy fruits for extended periods after maturation. Regarding the functional significance, fruiting calyces establish a physicochemical shield that reduces water loss and enables fruit maturation within a protective microclimate, and promotes different seed dispersal strategies among plant species investigated.

## Introduction

As different as organ functions of higher plants are, as different the requirements according to their surface properties turn out to be. Land plants have developed numerous adaptations to increase their performance and minimize the adverse factors imposed by living in a desiccating atmospheric environment (Huot et al., 2013). The plant cuticle represents a boundary layer between the plant and its surrounding environment covering extracellularly the outermost epidermal cell walls of aerial plant organs (Martin and Rose, 2014). This hydrophobic barrier limits uncontrolled water loss to the atmosphere and contributes to physical stabilization, reflects, absorbs, and transmits solar radiation, reduces pathogen infections, and offers resistance against herbivory (da Luz, 2006; Kosma et al., 2010; Petit et al., 2014).

Cuticular properties can vary enormously across plant species in an organ- and tissue-specific manner (Matzke and Riederer, 1991; Bonaventure et al., 2004; Jetter et al., 2006; Xu et al., 2014; Diarte et al., 2021). The structurally and compositionally heterogeneous plant cuticle can be subdivided into two distinct components: the cutin matrix and the cuticular waxes. Cuticular waxes are complex mixtures typically composed of homologous series of very-long-chain alkanoic acids, alkanals, alkanes, alkanones, alkanols, and alkyl esters. Along with these aliphatic compounds with chain lengths from 20 to 60 carbon atoms, tetracyclic sterols, and highly variable amounts of pentacyclic triterpenoids occur in cuticular waxes (Jetter et al., 2006; Samuels et al., 2008). By sealing the cutin matrix, cuticular waxes provide the main barrier to non-stomatal water loss (Riederer and Schreiber, 2001; Schreiber, 2010; Yeats and Rose, 2013), whereas the polymeric cutin matrix forms the structural framework of the cuticle and thus, substantially contributes to the physical integrity of plant organs. Characteristic cutin monomers are ω-hydroxyalkanoic acids with mid-chain epoxy, oxo, and hydroxy groups, and, infrequently, α,ω-dicarboxylic acids with chain lengths of 16 and 18 carbon atoms along with minor proportions of hydroxycinnamic acids. These long-chain aliphatic and aromatic cutin monomers are esterified into a complex network by primary and secondary ester bonds (Holloway, 1982; Fich et al., 2016; Leide et al., 2020).

Of exceptional interest in this context are cuticular barrier properties of plant species bearing fruiting calyces, which also serve as a protective and supportive envelope of fruits (Knapp, 2002; Wilf et al., 2017; Li et al., 2019). Fruiting calyces have evolved repeatedly across angiosperm clades, even though it is best known from the Solanaceae family. The evolution and development of this unique morphological feature have been comprehensively studied (He et al., 2004; He and Saedler, 2005; Deanna et al., 2017). For example, across the Solanoideae subfamily, the fruiting calyx morphology ranges from a non-accrescent calyx that does not enlarge during fruit maturation, and an accrescent but still appressed calyx to a highly inflated calyx (Hu and Saedler, 2007; Zamora-Tavares et al., 2016; Deanna et al., 2019). Such post-floral calyx morphogenesis is known as the inflated calyx syndrome, which causes persistent sepals to increase and inflate after flower fertilization (He and Saedler, 2007; Wilf et al., 2017; Li et al., 2019).

**Figure 1:**
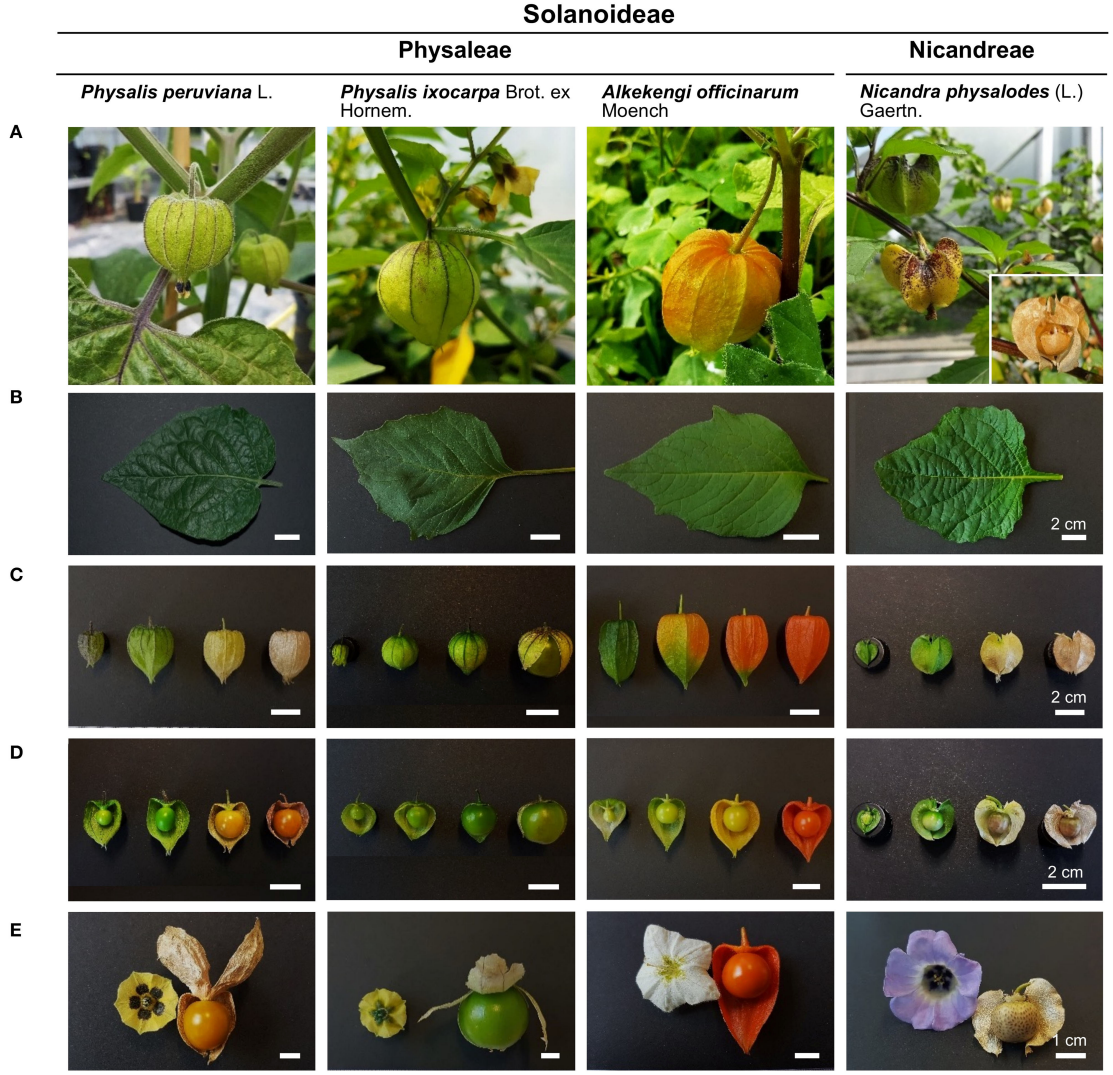
Morphological characteristics of leaves, fruiting calyces, and fruits of *Physalis, Alkekengi*, and *Nicandra* species belonging to the Physaleae and the Nicandreae tribe of the Solanoideae subfamily **(A)**. Fully expanded leaves are simple, ovate or ovate-elliptic **(B)**. Persistent sepals of the inflated fruiting calyces **(C)** change in extent, architecture, and color during fruit maturation **(D)**. Yellow, white, or blue petals form a tube with terminal lobes or teeth. Ripe fruits represent globose berries that bear a five-lobed fruiting calyx **(E)**.

Our study focused on the herbaceous to fruticose plant species *Physalis peruviana, Physalis ixocarpa*, and *Nicandra physalodes*, originally native to tropical America, and on *Alkekengi officinarum*, native to Asia, (Hu and Saedler, 2007). Their globose berry fruits hanging from the stem are enclosed with a characteristic pedicellate, five-lobed fruiting calyx (Horton, 1979; Knapp, 2002; Silva and Agra, 2005; Ortiz-Ramírez et al., 2018). Initially, the calyx of these plant species is a green, photosynthetic active whorl of sepals that strongly resembles leaves. During fruit maturation, the fruiting calyx expands and inflates, enveloping the fruit entirely. Fruits of *P. peruviana* and *N. physalodes* develop within an inflated fruiting calyx that becomes yellow and dries out at the late developmental stage. *P. ixocarpa* has a green or green-purple inflated fruiting calyx that bursts during the late phase of fruit expansion. The inflated fruiting calyx of *A. officinarum* becomes orange and, later, bright red and dries out. The ripe fruits of *A. officinarum* are bright red as well. At the ripe stage, fruits of *P. peruviana* and *P. ixocarpa* are orange and green or, infrequently, green-purple. In general, ripe *Physalis* and *Alkekengi* fruits are glossy and fleshy, whereas the green to yellow *N. physalodes* fruit dries out entirely during the ripening process, becoming a dull brownish fruit (Figure 1).

The inflated calyx syndrome as a morphological feature is well discussed in the literature, yet its post-floral functionality has been rarely investigated, and information about its potentially adaptive functioning is still lacking (Wilf et al., 2017; Li et al., 2019). Moreover, despite the increase in our understanding of the fruit cuticle-related properties over the past decade (Lara et al., 2014; Domínguez et al., 2017; Petit et al., 2017), only a few studies on the leaf cuticle of the Solanoideae subfamily were published so far, and comparative studies of the cuticular properties in different plant organs of the same plant species are still scarce (Fernández et al., 2016; Huang et al., 2017). Thus, this study aimed to investigate comprehensively the cuticle of phylogenetically closely related plant species bearing characteristic inflated fruiting calyces and to better understand the contribution of this morphological structure in determining the cuticular barrier profiles of *P. peruviana, P. ixocarpa, A. officinarum*, and *N. physalodes*. In particular, commonalities of leaves and characteristics of fruiting calyces at the cuticular level were investigated. Furthermore, we expected that fruits enclosed by a protective fruiting calyx possess unique cuticular barrier properties compared to leaves—at an organ-specific level—and to fruits of other solanaceous plant species—at a species-specific level.

## Materials and Methods

### Plant Material

*Physalis peruviana* L., *Physalis ixocarpa* Brot. ex Hornem., *Alkekengi officinarum* Moench and *Nicandra physalodes* (L.) Gaertn. plants were cultivated in the greenhouse. Plants were irrigated according to requirements and fertilized with Osmocote Plus (ICL Specialty Fertilizers) and Hakaphos Blau (Compo Expert). Fully expanded leaves, fully inflated fruiting calyces, and fruits at the fully ripe stage were investigated.

### Determination of Surface Area, Dry Weight, and Relative Water Content

Surface areas of leaves and fruiting calyces without petiole were determined according to the pixel values of the planar surface scanned at high resolution (600 dpi) and related to a reference area (Corel PHOTO-PAINT 2018). For that, calyces were cut open with the help of a scissor, and cuts were made on their sides, which made their opening and planning possible. They were kept planar on the scanner with the help of a projector transparent film. Fruits' surface areas were calculated from the average of their vertical and horizontal diameter. After fresh weight measurement, leaves, fruiting calyces, and fruits were oven-dried at 60°C for 7 days, and, afterward, the dry weight was measured to calculate their relative water contents (Sartorius MC-1 AC210S).

### Determination of Minimum Water Conductance and Cuticular Water Permeability

The minimum water conductance represents the lowest, unavoidable water loss of leaves and fruiting calyces with maximally closed stomata. It was determined gravimetrically from the consecutive weight loss of transpiring leaves and fruiting calyces in darkness at low atmospheric humidity (Diarte et al., 2021). The cuticular water permeability was determined gravimetrically from the transpiration of astomatous fruits under the same conditions (Leide et al., 2007). To access the post-floral contribution of the inflated calyx to the water loss of fruits, the cuticular water permeability was measured in ripe fruits with and without the fruiting calyx.

Weight loss of dehydrating leaves, fruiting calyces, and fruits was monitored over time using a high precision balance (Sartorius MC-1 AC210S) at 25°C. Cut petioles and peduncles were sealed with high-melting paraffin (Roth). The transpiration rate was calculated from the decline in weight over time and the total surface area. The water permeability was obtained by dividing the transpiration rate by the driving force for water loss. The driving force for vapor-based water loss was defined by the difference between the saturation concentrations in epidermal cells at the actual temperature and the surrounding atmosphere multiplied by the water activity in the epidermal apoplast or the atmosphere. The water activity of the atmosphere over silica gel (AppliChem) was nearly zero. The water activity in the epidermal apoplast was assumed to be close to one (Schuster et al., 2017). Thus, the active driving force was the saturation concentration of water vapor at the actual leaf, fruiting calyx, and fruit temperature. Corresponding, temperature-depending saturation concentration of water vapor was taken from Nobel (2009). Temperature and atmospheric humidity were monitored at intervals of 30 min using a digital thermo-hygrometer (Testoterm 6010).

### Cuticular Membrane Isolation

Cuticular membranes of fruits were enzymatically isolated with 1% (v/v) pectinase (Trenolin SuperPlus, Erbslöh), and 1% (v/v) cellulase (Celluclast, Novo Nordisk) in 20 mM citric acid (Roth), pH 3.0, containing 1 mM sodium azide (Sigma-Aldrich) at room temperature. The enzyme solution was exchanged weekly. Isolated cuticular membranes were extensively washed in deionized water and air-dried.

### Scanning Electron Microscopy

Air-dried leaves, fruiting calyces, and enzymatically isolated fruit cuticles were mounted on aluminum holders using conductive double-sided adhesive tape (Plano). Cross-sections of enzymatically isolated fruit cuticles were additionally mounted on aluminum holders for the measurement of cuticular thickness. Surfaces were sputter-coated with 10 nm to 15 nm gold and palladium (80/20) at 25 mA for 150 s (partial argon pressure 0.05 mbar; Balzers Bal-Tec SCD 005) and examined with a field emission scanning electron microscope (JEOL JSM-7500F) at 5 kV acceleration voltage and a 10 nm working distance.

### Cuticular Wax Extraction and Analysis by Gas Chromatography

Cuticular waxes of leaves and fruiting calyces were extracted with trichloromethane (Roth) at room temperature for 1 min and enzymatically isolated fruit cuticles for 5 min. For gas chromatographic analysis, *n*-tetracosane (C_24_; Fluka) was added as an internal standard. Afterward, the extracted cuticular waxes were dried under a continuous nitrogen flow until complete dryness and derivatized with *N,O*-bis-trimethylsilyl-trifluoroacetamide (Macherey-Nagel) in pyridine (Merck; 1/1, v/v) at 70°C for 60 min.

For quantification and identification of cuticular wax compounds, the extracts were diluted with trichloromethane and injected in a gas chromatograph (7890A, Agilent Technologies) coupled to a flame ionization detector and in a gas chromatograph (Agilent Technologies 6890N Network GC system) coupled to a mass spectrometer (5977A MSD, Agilent Technologies) in on-column mode. Capillary columns (Agilent J&W DB-1HT, 30 m length × 0.32 mm diameter × 0.1 μm film) were used, and hydrogen or helium as carrier gas. Gas inlet pressure was programmed at 50 kPa for 5 min, 3 kPa min^−1^ to 150 kPa, and at 150 kPa for 40 min. Temperature-programmed gas chromatographic conditions consisted of injection at 50°C for 2 min, then, raised by 40°C min^−1^ to 200°C, holding for 2 min at 200°C, subsequently, increased by 3°C min^−1^ to 320°C, and, finally, held at 320°C for 30 min. Cuticular waxes were identified by the electron ionization-mass spectra.

The distribution of carbon chain lengths was calculated as the average carbon chain lengths, defined as


ACL = Σ (Cn × n)/Σ (Cn)

where C*n* was the abundance of aliphatic moieties with *n* carbon units.

### Cutin Transesterification, Extraction, and Analysis by Gas Chromatography

The enzymatically isolated and dewaxed fruit cuticle was transesterified with boron trifluoride in methanol (Sigma-Aldrich) at 70°C overnight. After cooling, *n*-dotriacontane (C_32_; Sigma-Aldrich) as internal standard and trichloromethane (1:1, v/v; Roth) were added to the extracts. Transmethylated cutin monomers were washed three times with sodium-chloride saturated aqueous solution, dried with anhydrous sodium sulfate (AppliChem), and filtered. Afterward, the organic solvent was evaporated under a gentle flow of nitrogen. Derivatization with *N,O*-bis-trimethylsilyl-trifluoroacetamide in pyridine was performed at 70°C for 60 min.

Separation of cutin monomers was carried out at 50 kPa for 60 min, 10 kPa min^−1^ to 150 kPa, and at 150 kPa for 30 min using a temperature program of 50°C for 1 min, raised by 10°C min^−1^ to 150°C, held at 150°C for 2 min, and, subsequently, increased by 3°C min^−1^ to 320°C and maintained at 320°C for 30 min. The qualitative and quantitative composition was studied using capillary gas chromatography with mass spectrometric and flame ionization detection under the same chromatographic conditions.

### Statistical Analysis

Data were compared by analysis of variance followed by an HSD *post-hoc* test for unequal *n* (Dell Statistica 13.1). *p* values <0.05 were considered significantly different.

## Results

### Micromorphology of Leaves, Fruiting Calyces, and Fruits

**Figure 2:**
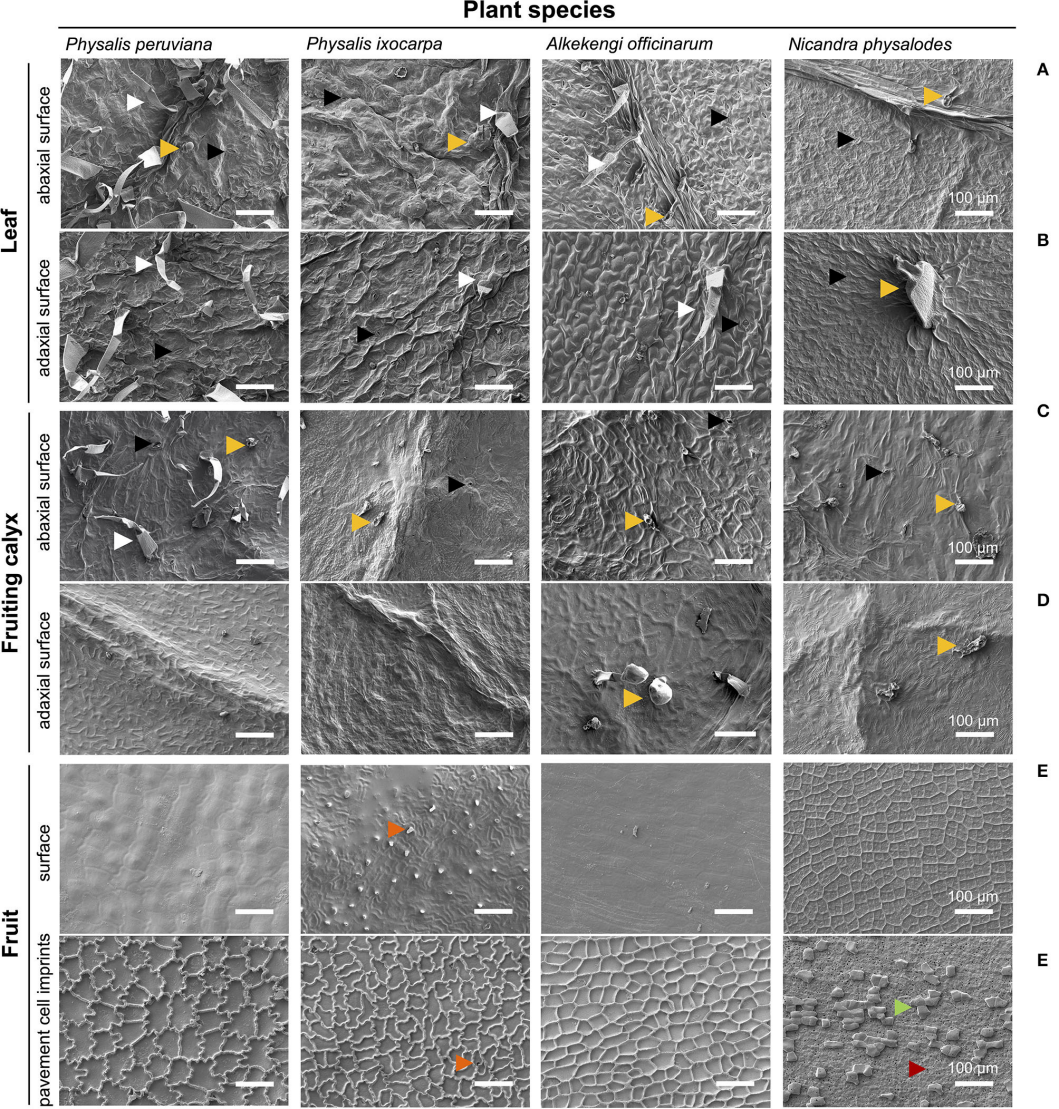
Micromorphological characteristics of the leaf **(A,B)**, the fruiting calyx **(C,D)**, and the fruit surface **(E,F)** of *Physalis, Alkekengi*, and *Nicandra* species. Scanning electron microscopy was utilized for examining the abaxial and adaxial sides of fully expanded leaves and fully inflated fruiting calyces. Using enzymatically isolated fruit cuticles, the surface of ripe fruits and, on its reverse side, imprints of pavement cells were scanned. Arrowheads indicate stomata (black), non-glandular trichomes (white), glandular trichomes (light orange), cuticular protuberances/invaginations (dark orange), sclerified cells (green), and epidermal pores (red).

At the microscopic scale, fully expanded leaves of *P. peruviana, P. ixocarpa, A. officinarum*, and *N. physalodes* were similar having non-glandular and/or glandular trichomes besides stomata on the abaxial and the adaxial surface (amphistomatous) with side -and species-specific distributions (Figures 2A,B). Fully inflated fruiting calyces mainly exhibited glandular trichomes, and stomata were only detected on the abaxial surface (hypostomatous, Figures 2C,D). Exclusively, *P. peruviana* bore non-glandular and glandular trichomes on the abaxial fruiting calyx surface. An examination of the microstructure revealed an epicuticular wax film and, intermittently, irregular epicuticular wax granules covering the abaxial and the adaxial surface of leaves and fruiting calyces (Figures 2A–D, Supplementary Figures S1A–D).

The surface of ripe fruits exhibited neither trichomes nor stomata (astomatous) but the fruit surface of *P. ixocarpa* had cuticular protuberances (Figure 2E). Fruit surfaces of *Physalis* and *Alkekengi* species were covered by a smooth epicuticular wax film with few irregular epicuticular wax granules. Possessing hardly any cuticular waxes, the concave epidermal cell structure of *N. physalodes* fruits emerged (Supplementary Figure S1E). The shape and extent of the outermost epidermal cells (pavement cells) of fruits were visualized by their cuticular imprints showing in *Physalis* an intricate, undulate structure of anticlinal cell walls of different dimensions. The anticlinal cell walls of *A. officinarum* epidermal cells had an alveolate structure. Micrographs of *Physalis* and *Alkekengi* fruits highlighted the continuous cuticularisation of the outer periclinal, epidermal cell wall. In contrast, the cuticular imprints of *N. physalodes* were masked with a layer of sclerified cells perforated with epidermal pores (Figure 2F, Supplementary Figure S1F).

### Cuticular Waxes of Leaves, Fruiting Calyces, and Fruits

**Figure 3:**
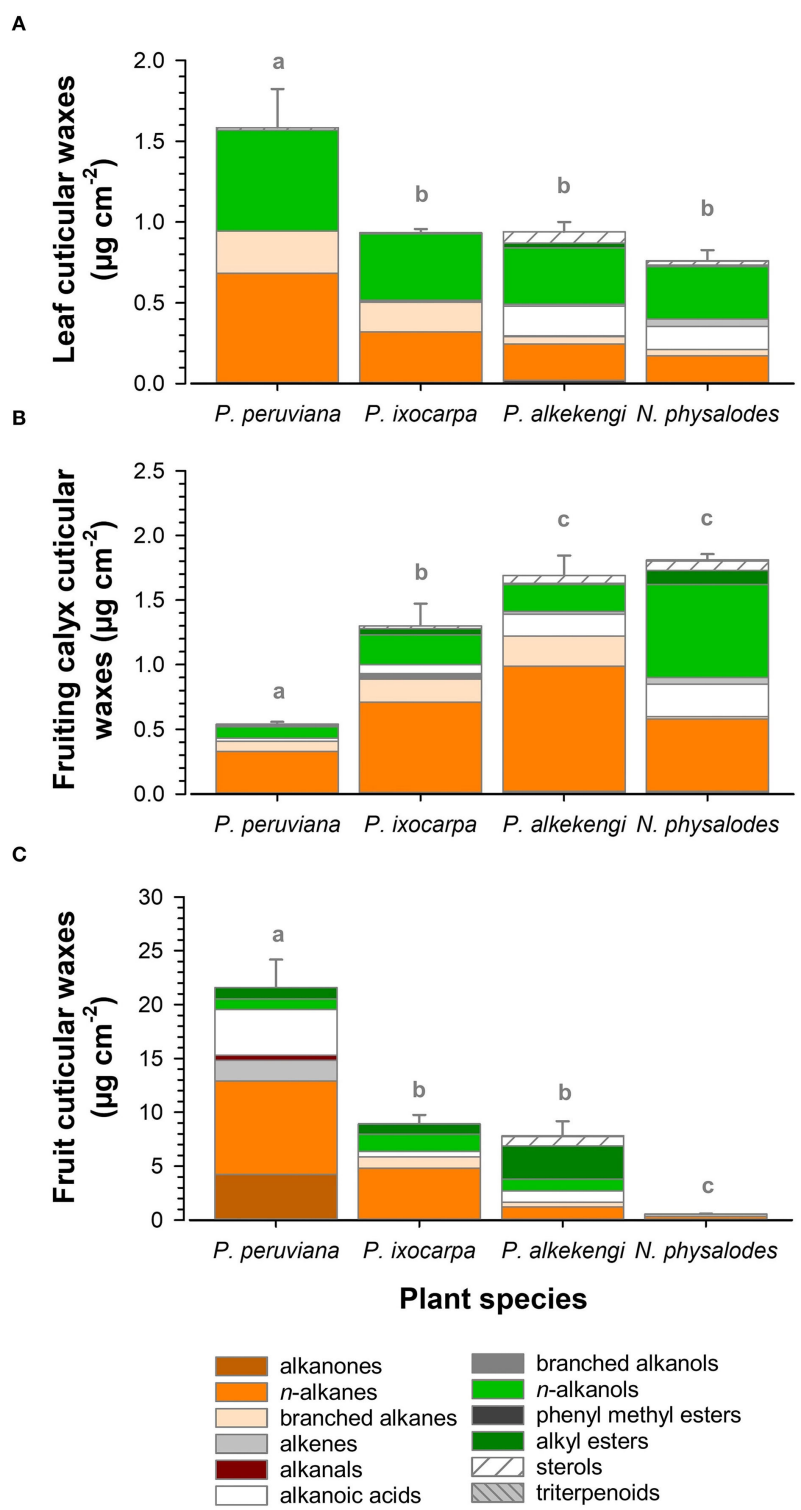
Compositional analysis of cuticular wax load of fully expanded leaves **(A)**, fully inflated fruiting calyces **(B)**, and ripe fruits **(C)** of *Physalis, Alkekengi*, and *Nicandra* species subdivided into compound classes. Data are shown as mean ± SD (*n* ≥ 4). Different letters indicate significant differences between plant species (*p* < 0.05).

Cuticular wax deposition of *P. ixocarpa, A. officinarum*, and *N. physalodes* were about 0.9 μg cm^−2^ on fully expanded leaves and 1.6 μg cm^−2^ on fully inflated fruiting calyces, respectively (Figures 3A,B). Exclusively, *P. peruviana* leaves almost doubled the cuticular wax accumulation with 1.6 μg cm^−2^, whereas the corresponding fruiting calyces of *P. peruviana* with 0.5 μg cm^−2^ had at least a threefold reduction of the cuticular wax load. Very-long-chain aliphatic compounds dominated the cuticular wax loads of leaves and fruiting calyces (≥93% of total cuticular waxes).

**Figure 4:**
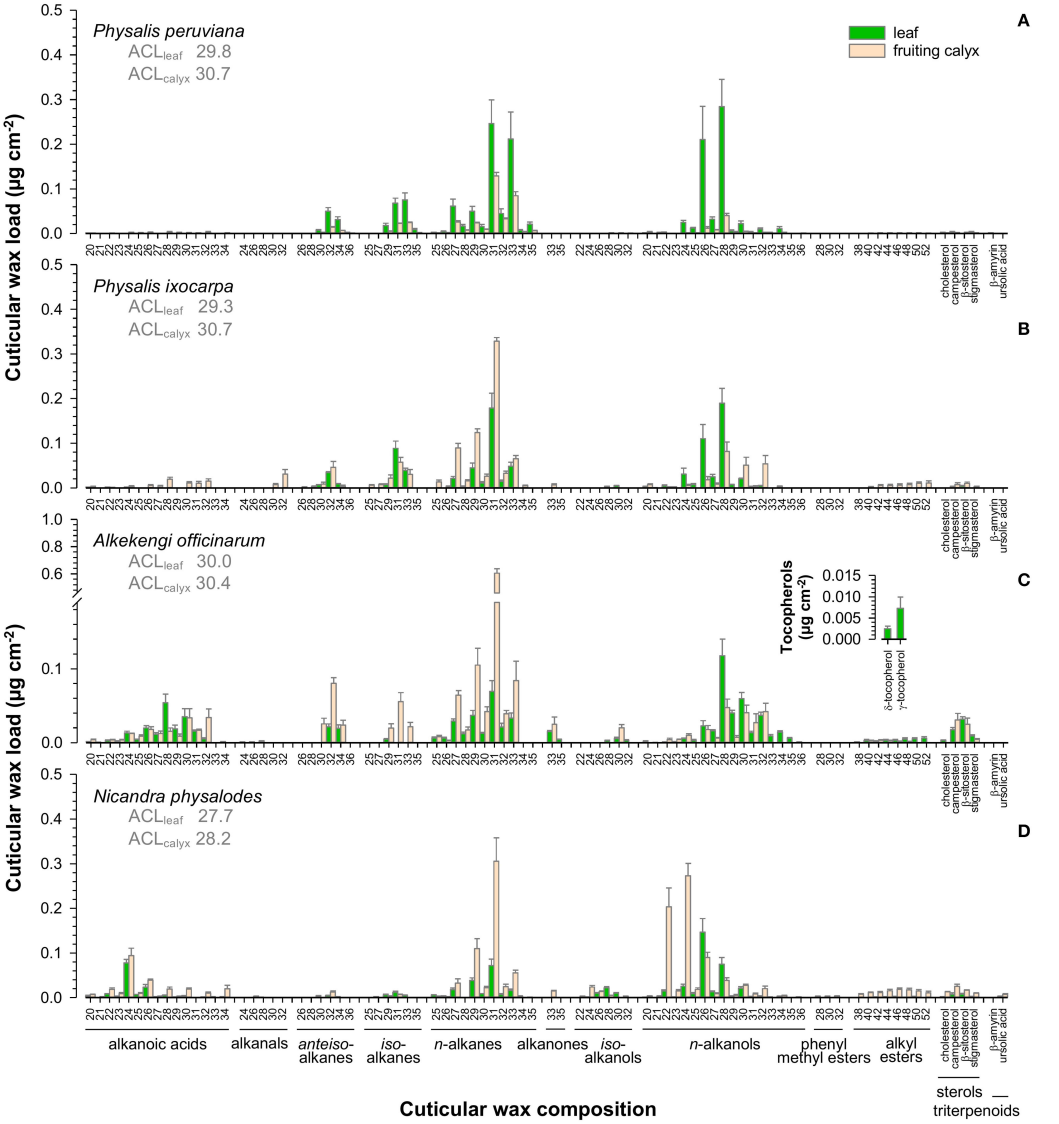
Gas chromatographic analysis of the cuticular wax composition of *Physalis ixocarpa* **(A)**, *Physalis peruviana* **(B)**, *Alkekengi officinarum* **(C)**, and *Nicandra physalodes* **(D)** fully expanded leaves and fully inflated fruiting calyces. Average carbon chain lengths calculated for the aliphatic wax fraction are given (ACL). Data are shown as mean ± SD (*n* ≥ 4).

Aliphatic wax fractions of leaves mainly consisted of *n*-alkanes and *n*-alkanols, most prominently nonacosane (C_29_), hentriacontane (C_31_), tritriacontane (C_33_) and hexacosanol (C_26_), octacosanol (C_28_), triacontanol (C_30_; Figure 4). Moreover, cuticular waxes contained high amounts of *iso*- and *anteiso*-branched alkanes on *Physalis* leaves (Figures 4A,B), and alkanoic acids on *A. officinarum* and *N. physalodes* leaves (Figures 4C,D). In small quantities, alkanals, alkanones, *iso*-branched alkanols, phenyl methyl esters, alkyl esters, tetracyclic sterols, and pentacyclic triterpenoids were infrequently detected in leaf cuticular waxes. In addition, tocopherols were found admixed to the cuticular waxes of *A. officinarum* leaves.

*N*-alkanes, most notably hentriacontane (C_31_), *n*-alkanols, and alkanoic acids, constituted the major compound classes of fruiting calyx cuticular waxes (Figure 4). Additionally, cuticular waxes of *Physalis* and *Alkekengi* fruiting calyces exhibited high amounts of *iso*- and *anteiso*-branched alkanes. Infrequently, alkanals, alkanones, *iso*-branched alkanols, alkyl esters, tetracyclic sterols, and pentacyclic triterpenoids were found in minor amounts on fruiting calyces.

Despite similar compound class preferences, different carbon chain length distributions were detected for leaves and fruiting calyces. Average carbon chain lengths ranged from 27.7 to 30.7, with leaves having lower values than the corresponding fruiting calyces. A species-specific comparison exhibited the lowest average length of carbon chains for *N. physalodes* leaves and fruiting calyces compared to *Physalis* and *Alkekengi* species.

Comparing the cuticular wax coverages, the compositional diversity was the highest on ripe fruits compared to fully expanded leaves and fully inflated fruiting calyces. Amounts of total fruit cuticular waxes ranged widely from 0.7 μg cm^−2^ for *N. physalodes* to 21.6 μg cm^−2^ for *P. peruviana* (Figure 3C). Thus, ripe *Physalis* and *Alkekengi* fruits had at least tenfold higher cuticular wax loads than their corresponding leaves at an organ-specific level. *N. physalodes* fruits exhibited a cuticular wax coverage comparable to leaves.

**Figure 5:**
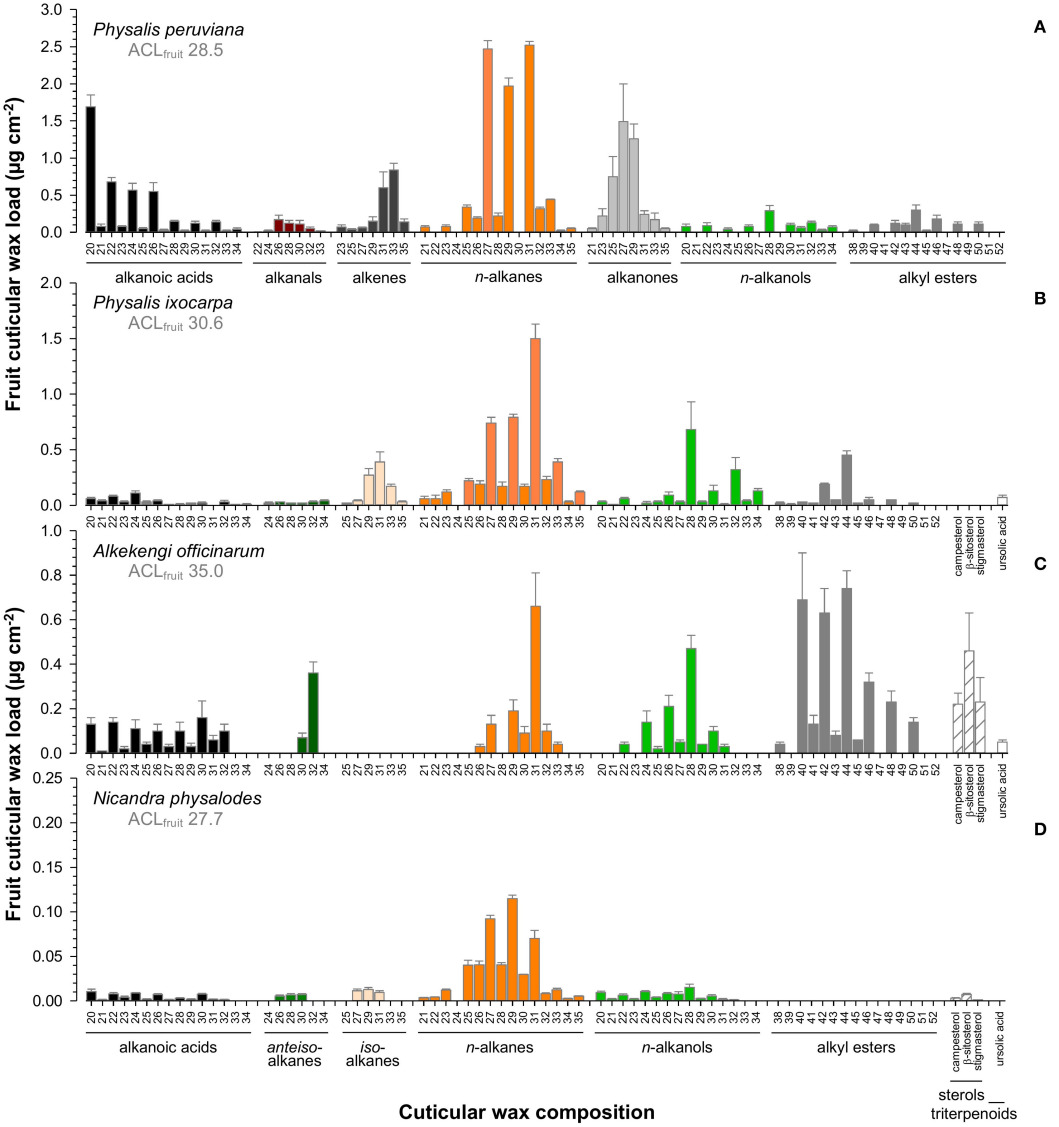
Gas chromatographic analysis of the cuticular wax composition of *Physalis ixocarpa* **(A)**, *Physalis peruviana* **(B)**, *Alkekengi officinarum* **(C)**, and *Nicandra physalodes* **(D)** ripe fruits. Average carbon chain lengths calculated for the aliphatic wax fraction are given (ACL). Data are shown as mean ± SD (*n* ≥ 4).

Fruit cuticular waxes were mainly composed of very-long-chain aliphatic compounds with ≥98% of total cuticular waxes (Figures 5A–D) except for *A. officinarum* fruits, in which cuticular waxes comprised 12% tetracyclic sterols (campesterol, β-sitosterol, stigmasterol, Figure 5C). Moreover, the predominance of *n*-alkanes, *iso-*, and *anteiso*-branched alkanes, *n*-alkanols, and alkanoic acids in cuticular waxes held only true for *P. ixocarpa* and *N. physalodes* fruits (Figures 5B,D). Cuticular waxes of *Physalis* and *Alkekengi* fruits were also characterized by substantial quantities of alkyl esters, particularly in the case of *A. officinarum*. Fruit cuticular waxes of *A. officinarum* were dominated by alkyl esters (39% of total cuticular waxes) mainly with a chain length of 40, 42, and 44 carbon units. Conversely, cuticular waxes of *P. peruviana* fruits comprised high amounts of alkanals, alkenes, and alkanones (31% of total cuticular waxes) but *iso*- and *anteiso*-branched alkanes were not detected (Figure 5A). Hence, average carbon chain lengths calculated for the very-long-chain aliphatic wax fraction of fruits amounted to between 27.7 for *N. physalodes* and 35 for *A. officinarum*.

### Compositional Characteristics of Cutin Matrices of Fruits

**Figure 6:**
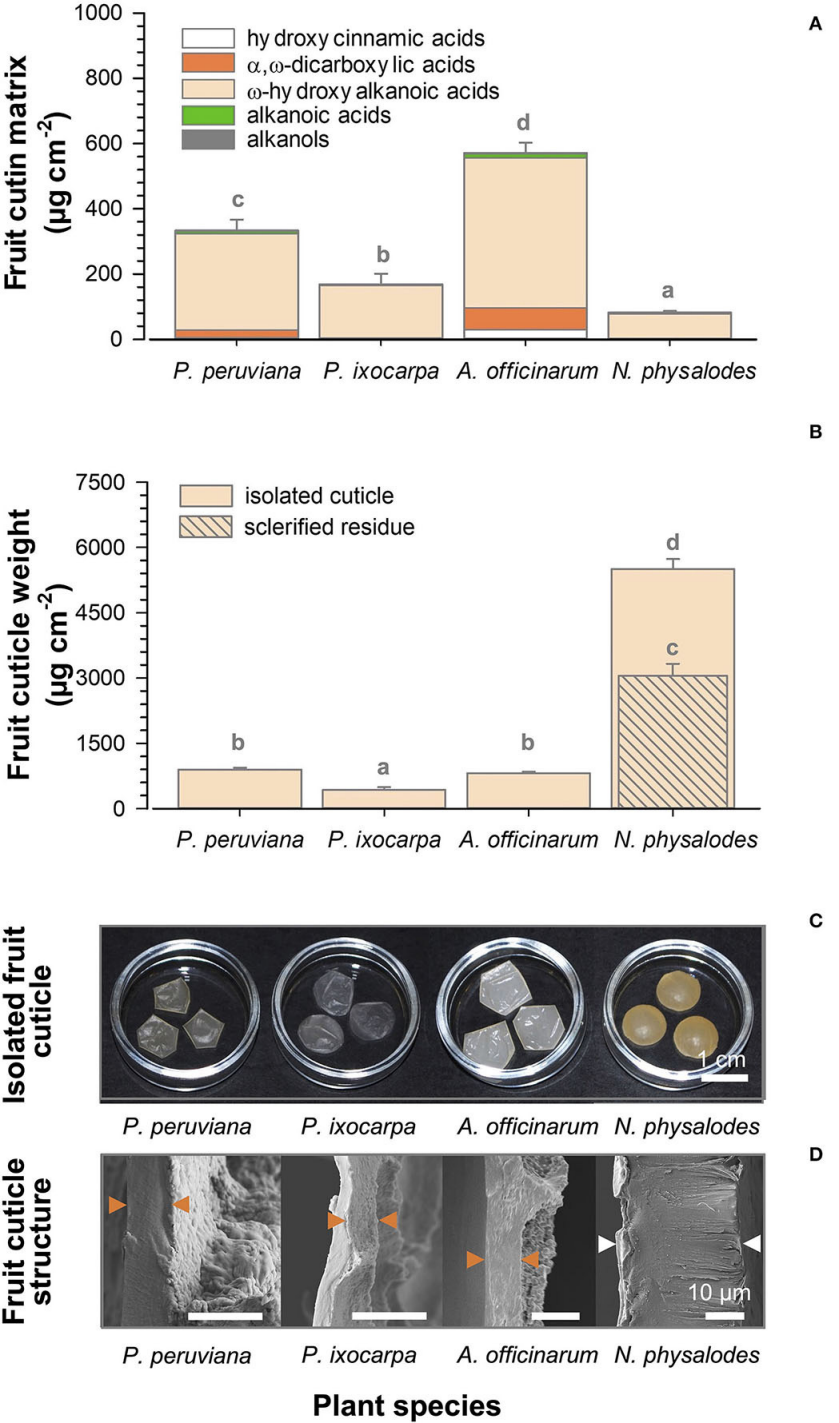
Gas chromatographic analysis of cutin deposition of *Physalis, Alkekengi*, and *Nicandra* ripe fruits subdivided into compound classes of cutin monomers **(A)**. Gravimetric data of enzymatically isolated fruit cuticle and the non-extractable, non-hydrolysable residue **(B)**. Data are shown as mean ± SD (*n* ≥ 4). Different letters indicate significant differences between plant species (*p* < 0.05). Photographs of enzymatically isolated fruit cuticle **(C)**. Scanning electron micrographs of cross-sections of isolated fruit cuticles. Arrowheads indicate the isolated fruit cuticle (orange) or the isolated fruit cuticle plus a layer of sclerified cells (white; **D**).

Cutin depositions on ripe fruit surfaces were quantified between 81.4 μg cm^−2^ for *N. physalodes* and 570.7 μg cm^−2^ for *A. officinarum* (Figure 6A). Despite these substantial quantitative differences, the cutin matrix commonly consisted of ω-hydroxyalkanoic acids, α,ω-dicarboxylic acids, alkanoic acids, alkanols, and hydroxycinnamic acids in different relative contributions. The proportion of ω-hydroxyalkanoic acids ranged from 89 to 96% of total cutin monomers of *P. peruviana, P. ixocarpa*, and *N. physalodes* fruits. However, it amounted only to 81% on *A. officinarum* fruits in which the proportion of α,ω-dicarboxylic acids, and hydroxycinnamic acids esterified within the cutin matrix was enhanced to 12 and 5%, respectively.

**Figure 7:**
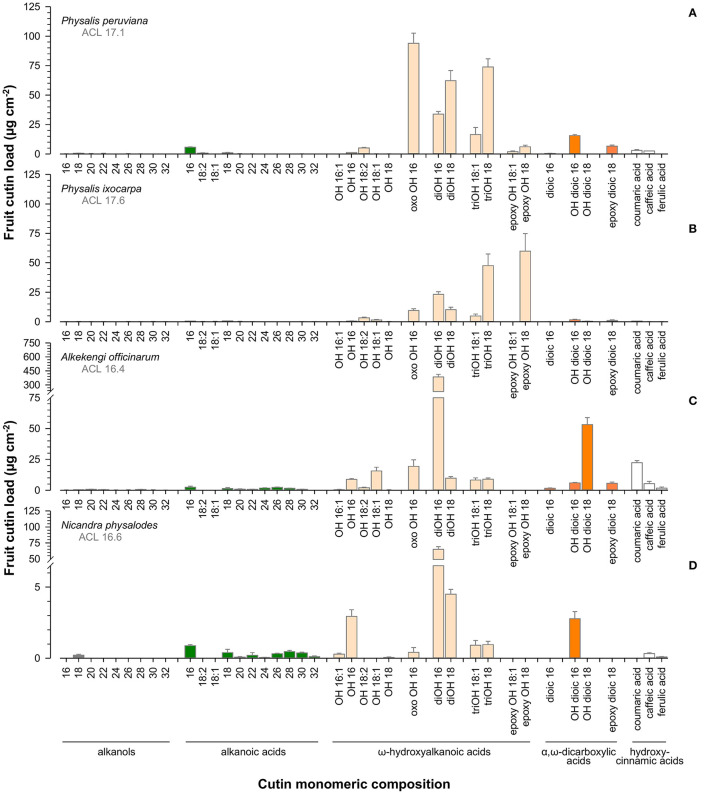
Gas chromatographic analysis of cutin monomeric composition of *Physalis ixocarpa* **(A)**, *Physalis peruviana* **(B)**, *Alkekengi officinarum* **(C)**, and *Nicandra physalodes* **(D)** ripe fruits. Average carbon chain lengths calculated for the aliphatic cutin monomers are given (ACL). Data are shown as mean ± SD (*n* ≥ 4).

Aliphatic cutin monomers had a chain length distribution between 16 and 32 carbon units distinctly dominated by ω-hydroxyhexadecanoic (C_16_) and ω-hydroxyoctadecanoic acids (C_18_; Figure 7). Principal aliphatic cutin monomers were 9/10-oxo-ω-hydroxyhexadecanoic, 9/10,ω-dihydroxyoctadecanoic, and 9,10,ω-trihydroxyoctadecanoic acids for *P. peruviana* (Figure 7A), 9,10-epoxy-ω-hydroxyoctadecanoic and 9,10,ω-trihydroxyoctadecanoic acids for *P. ixocarpa* (Figure 7B) but 9/10,ω-dihydroxyhexadecanoic acid for *A. officinarum* (Figure 7C) and *N. physalodes* (Figure 7D) in different quantities. Mainly resulting from different principal cutin monomers, a species-specific carbon chain length distribution of about 16.5 for *A. officinarum* and *N. physalodes* to 17.6 for *P. ixocarpa* fruit cutin was calculated. The cutin matrix of *Physalis* and *Alkekengi* fruits exhibited coumaric acid as a principal aromatic cutin monomer that was not detected for *N. physalodes*, which fruits possessed a comparatively high abundance of caffeic acid.

### Cuticle Weight, Cuticle Thickness, and Cuticle Structure of Fruits

Weights of the enzymatically isolated cuticle of ripe fruits were gravimetrically determined. The lowest cuticle weight per area was found for *P. ixocarpa* with 424.9 μg cm^−2^ (Figures 6B,C). Cuticle weights of *P. peruviana* and *A. officinarum* fruits were twofold higher. Uniquely, *N. physalodes* fruits had a cuticle that was enzymatically not isolable from a subjacent layer of sclerified cells. The cuticle of *N. physalodes* plus the sclerified layer amounted to 5499.5 μg cm^−2^. Thus, a non-extractable, non-hydrolysable sclerified residue of 3058.2 μg cm^−2^ remained after cuticular wax extraction and cutin depolymerisation of the *N. physalodes* fruit cuticle.

The thickness of the cuticle covering the outermost periclinal cell walls was 6.3 μm for *P. peruviana*, 4.2 μm for *P. ixocarpa*, and 8.6 μm for *A. officinarum* fruits. Cuticular pegs pointed to a cuticular thickening between the anticlinal epidermal cell walls. A very thin cuticle was found on ripe fruits of *N. physalodes* cross-linked to an extremely thick layer of elongated sclerified cells amounting together to 30.9 μm (Figure 6D).

### Functional Characteristics of Leaves, Fruiting Calyces, and Fruits

**Figure 8:**
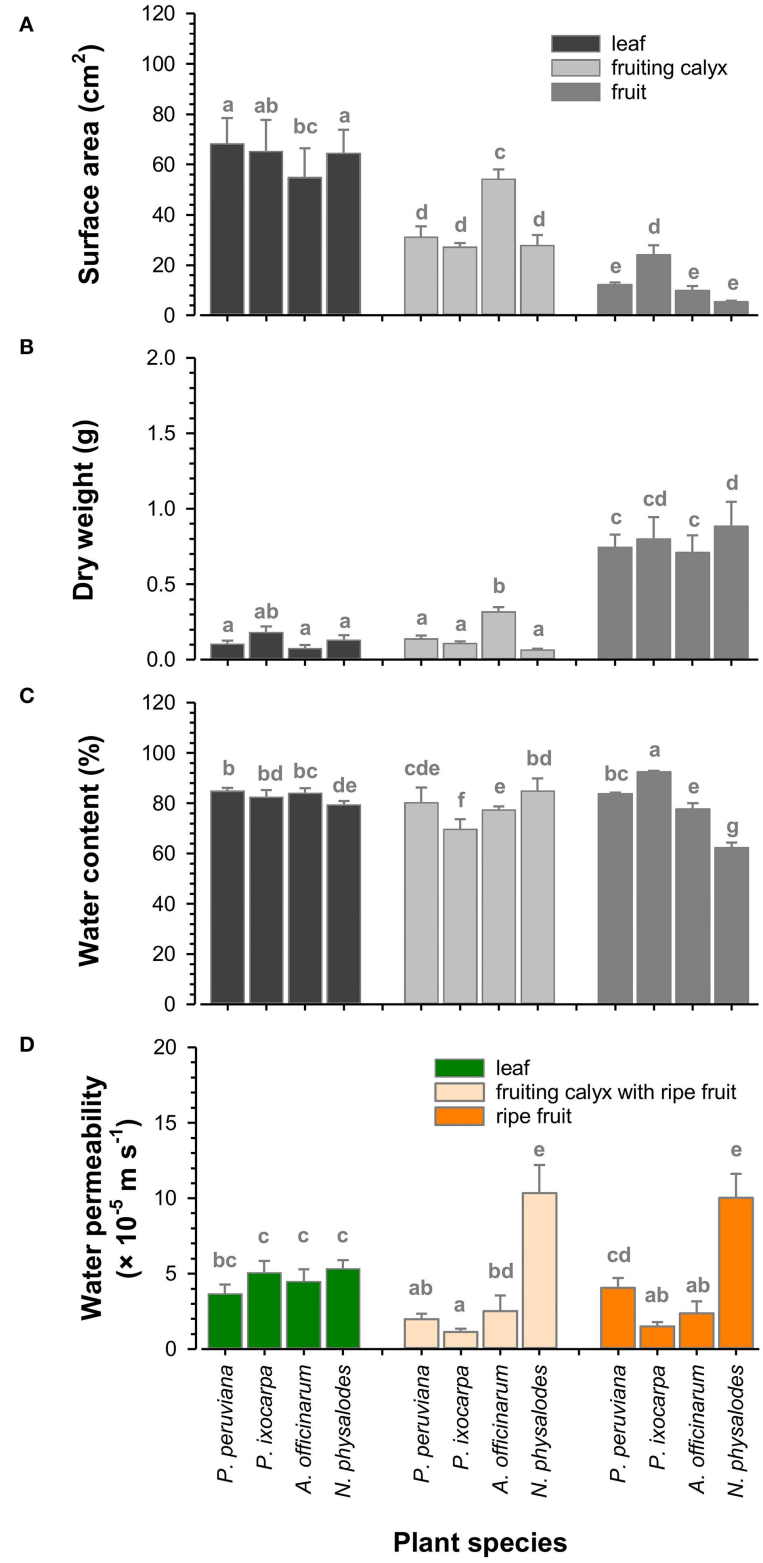
Functional characteristics of *Physalis, Alkekengi*, and *Nicandra species*. Total surface area **(A)**, dry weight **(B)**, and relative water content **(C)** of fully expanded leaves, fully inflated fruiting calyces, and ripe fruits were measured. Minimum water conductance was investigated for leaves and fruiting calyces, including fruits. Cuticular water permeability was investigated for fruits without fruiting calyces **(D)**. Data are shown as mean ± SD (*n* ≥ 8). Different letters indicate significant differences (*p* < 0.05).

Fully expanded leaves were similar in total surface area of about 63.1 cm^2^ considering the adaxial and abaxial leaf sides (Figure 8A). Total surface areas of fully inflated fruiting calyces of *Physalis* and *Nicandra* species were comparable with about 28.7 cm^2^ but two times reduced compared to corresponding leaves. At an intraspecific level, fruiting calyces were almost as large as corresponding leaves, except for *A. officinarum*. Dry weights were about 0.1 g per leaf or fruiting calyx, with the lowest dry weight for leaves and the highest amount for fruiting calyces of *A. officinarum* (Figure 8B). The relative water content of leaves and fruiting calyces ranged between 77 and 85%. Only the relative water content of *P. ixocarpa* fruiting calyces was lower at 70% (Figure 8C).

However, the relative water content of ripe fruits was highly diverse (Figure 8C). The highest amount of 92% was found in *P. ixocarpa* fruits, which was vice versa enclosed by fruiting calyces with the lowest relative water content. Fruiting calyces and fruits of *P. peruviana* and *A*. officinarum had a similar relative water content averaging 80%. Still, it was distinctly reduced in *N. physalodes* fruits by 23% compared to corresponding fruiting calyces. *P. peruviana, A. officinarum*, and *N. physalodes* fruits had the smallest total surface area with leaves and fruiting calyces ranging between 5.3 and 12.1 cm^2^ (Figure 8A). Fruits were at least three times reduced in size compared to the total surface area of their enclosing fruiting calyces except for *P. ixocarpa* fruiting calyces and fruits having a total surface area of about 25.6 cm^2^ in common. Dry weights of fruits, including seeds, were about 0.8 g and, thus, doubled at least the dry weight of leaves and fruiting calyces (Figure 8B, Supplementary Figure S2).

The minimum water conductance of leaves was about 4.6 × 10^−5^ m s^−1^ (Figure 8D). The cuticular water permeability of *P. ixocarpa* and *A. officinarum* fruits was lower at about 1.9 × 10^−5^ m s^−1^. *P. peruviana* fruits had a comparable water permeability as leaves, whereas *N. physalodes* fruits doubled the leaf water permeability. *Physalis peruviana* fruits showed a significant twofold increase in water permeability after the fruiting calyx removal than the water permeability measured for its enclosed fruits. However, the water permeability of fruits with and without fruiting calyces remained at the same level for the other plant species.

## Discussion

### Commonalities of Solanoideae Leaves

Ovate or ovate-elliptic leaves of *Physalis, Alkekengi*, and *Nicandra* species were morphologically and functionally similar. Leaves contained about 83% water, and their minimum water conductance averaged at 5 × 10^−5^ m s^−1^ after maximum stomatal closure, thus comparable to *Solanum lycopersicum* leaves (Schuster et al., 2017). As commonly found in solanaceous plant species, *Physalis, Alkekengi*, and *Nicandra* leaves had different non-glandular and glandular trichomes (Ahmad, 1964; Dyki et al., 1998). For example, *P. peruviana* possessed trichome-rich leaves carrying most notably long non-glandular trichomes, whereas trichomes were only sparsely distributed on *N. physalodes* leaves. Trichomes comprise a physical and chemical coating of plant surfaces protecting, amongst others, against pathogen and herbivore attacks, being commonly found in solanaceous plant species (McDowell et al., 2011; Rodríguez-López et al., 2011; Lucatti et al., 2013).

Contrary to fruits, only a few studies on the leaf cuticle of the Solanoideae subfamily were published so far, and a still limited number of plant species, most crops, were investigated in terms of cuticle properties (Martin and Rose, 2014; Lara et al., 2015; Domínguez et al., 2017). In our study, cuticular wax loads of *Physalis, Alkekengi*, and *Nicandra* leaves ranged between 1 and 2 μg cm^−2^ and, thus, were as low as those found on leaves of *Capsicum annuum, Capsicum chinense, Solanum tuberosum*, and *S. lycopersicum*. Besides, cuticular wax profiles of *Physalis, Alkekengi*, and *Nicandra* leaves were characterized by a highly aliphatic nature as previously documented for other solanaceous leaves (Vogg et al., 2004; Guo and Jetter, 2017; Macel et al., 2020). Very-long-chain *n*-alkanes and *n*-alkanols dominated the cuticular waxes in addition to high amounts of *iso*- and *anteiso*-branched alkanes on *Physalis* leaves and alkanoic acids on *A. officinarum* and *N. physalodes* leaves. Notably, the abundance of *n*-alkanes and branched alkanes in cuticular waxes and their specific carbon chain length distribution are often considered species-specific attributes of solanaceous plant species (Haliński et al., 2017). Nevertheless, despite different cuticular wax amounts and variations in compound classes and carbon chain length distributions of very-long-chain aliphatic wax compounds, these quantitative and compositional modifications did not significantly affect the transpiration barrier properties of *Physalis, Alkekengi* and *Nicandra* leaves. Altogether, we were able to show commonalities in the leaf cuticle composition, leaf surface structure, and minimum conductance of leaves within the Solanoideae subfamily. These analyses provide further insights into leaf cuticle-related properties and highlight the potential of this unconventional group of plants for further studies on plant cuticular biology.

### Diversity of Solanoideae Fruits

Most distinguishable characteristics among solanaceous plant species are generally related to their reproductive plant organs (Knapp, 2002; Pabón-Mora and Litt, 2011). Appropriately, *Alkekengi* and *Nicandra* are largely used as ornamental plants, whereas *Physalis* species are primarily cultivated for their edible and nutritious fruits (Silva and Agra, 2005; Medina-Medrano et al., 2015).

Even though *Physalis, Alkekengi*, and *Nicandra* species have astomatous and globose berry fruits in common, the functional traits of their ripe fruits were considerably different regarding the surface area, relative water content, and cuticular water permeability. The water permeability of fleshy *Physalis* and *Alkekengi* fruits ranged from 2 × 10^−5^ to 4 × 10^−5^ m s^−1^ and was almost at the same level as *C. annuum* and *S. lycopersicum* fruits (Schreiber and Riederer, 1996; Vogg et al., 2004; Leide et al., 2007). Voluminous *P. ixocarpa* fruits with high relative water content had a distinctly low water permeability. On the other hand, *N. physalodes* bore small fruits with low relative water contents and, vice versa, an uncommonly high cuticular water permeability of 10 × 10^−5^ m s^−1^. Consequently, *N. physalodes* fruits were as dry as their enveloping calyces at the late maturation stage. Yet, the permeability values herein found for the solanaceous fruits, as well as for leaves, were within the range previously described in the literature (Schuster et al., 2017).

Micromorphologically, the surface of ripe *Physalis, Alkekengi*, and *Nicandra* fruits was characterized by the absence of stomata and trichomes, which made them distinguishable from trichome-rich solanaceous species, such as *S. lycopersicum, Solanum pennellii* and *Solanum habrochaites* fruits (McDowell et al., 2011). The lack of these defensive structures underlines the importance of the fruiting calyx as a physicochemical obstacle to reducing the susceptibility of its enclosed fruits to biotic and abiotic stresses. Interestingly, the fruit surface of *N. physalodes* had epidermal cells with a concave shape, resembling the surface observed in dry plant surfaces such as seeds, which can be a result of cell shrinkage due to the increased water loss showed by *Nicandra* fruits during maturation (Koch et al., 2008). *Physalis ixocarpa* was unique for being covered with papilla-like projections on the fruit surface, a feature also documented by Dyki et al. (1997). Papillate surfaces were also identified on fruits of *Anabasis* species and *Dysphania bhutanica* (Amaranthaceae; Sukhorukov, 2008, 2012).

Furthermore, it has been reported that cuticular waxes of solanaceous fruits typically comprising 2 to 15 μg cm^−2^ include large quantities of very-long-chain *n*-alkanes and pentacyclic triterpenoids, for example, *Solanum cheesmaniae, Solanum pimpinellifolium, S. lycopersicum, C. annuum, C. chinense* fruits (Vogg et al., 2004; Leide et al., 2007; Isaacson et al., 2009; Parsons et al., 2012; Yeats et al., 2012; Petit et al., 2014). Pentacyclic triterpenoids as cuticular wax fractions play multiple roles such as reducing pathogen susceptibility, attenuating harmful UV-radiation, and mechanically strengthening the cutin matrix but, contrary to very-long-chain aliphatic wax compounds, triterpenoids do not substantially contribute to cuticular water barrier properties (Szakiel et al., 2012; Tsubaki et al., 2013; Moses et al., 2015; Jetter and Riederer, 2016).

*Physalis, Alkekengi*, and *Nicandra* fruits were exceptionally positioned among these solanaceous fruits, accumulating typical amounts of cuticular waxes of about 8 μg cm^−2^ on *P. ixocarpa* and *A. officinarum* fruits but with a highly aliphatic character. Just a few solanaceous fruits with a small or an absent triterpenoid wax fraction as in solanaceous leaves are known but their cuticular wax loads were considerably higher. Yeats et al. (2012) showed that fruits of *Solanum neorickii* and *Solanum chmielewskii* are covered with >20 μg cm^−2^ cuticular waxes, as similarly found in *P. peruviana* fruits. However, in the latter case, the cuticular wax composition differed strikingly from a typical aliphatic cuticular wax pattern of solanaceous fruits containing largely *n*-alkanes, alkanols, alkanoic acids, and alkyl esters.

The high cuticular wax amount and the extraordinary cuticular wax composition of *P. peruviana* fruits might be the determining factor for its high cuticular water permeability, which was twice as high as the water permeability of *P. ixocarpa* and *A. officinarum* fruits. *N. physalodes* fruits additionally underlined the significance of cuticular waxes as the main barrier against transpirational water loss (Riederer and Schreiber, 2001; Leide et al., 2007), exhibiting a similar composition as *P. ixocarpa* and *A. officinarum* fruits but with a considerably lower amount at <1 μg cm^−2^, and a severe cuticular water loss. Consequently, differences in cuticular wax amounts and very-long-chain aliphatic compounds might have resulted in unequal cuticular barrier properties of *Physalis, Alkekengi*, and *Nicandra* fruits (Grncarevic and Radler, 1967; Bueno et al., 2019; Diarte et al., 2021).

*N. physalodes* fruits had a considerably higher cuticular water permeability, transpiring even more water than corresponding leaves, although both *N. physalodes* organs accumulated a similar cuticular wax coverage and composition. Isaacson et al. (2009) reported that cutin deficiency did not affect cuticular water permeability when analyzing *S. lycopersicum* fruits. Nevertheless, in this case, the polymeric cutin matrix, which significantly contributes to the structural integrity of the cuticle and, hence, provides fracture toughness to ripe fruits (López-Casado et al., 2007; Khanal and Knoche, 2017), was relatively thin deposited on the outer epidermal surface of *Nicandra* fruits. Altogether, the extremely high cuticular water loss rate of *Nicandra* fruits might be a physiological adaptation related to its seed dispersion strategy. Since no stomata were identified on fruits and were only sparsely present on the abaxial fruiting calyx surfaces, the desiccation process observed in *Nicandra* fruits might result from its distinct cuticular properties. The stiffness resulting from the sclerified cells combined with the low plasticity of the scarcely existent cutin matrix, plus the low cuticular wax amount, might contribute to the reduced waterproofing efficiency of the *N. physalodes* fruit surface. Due to the less effective cuticular transpiration barrier, the high water permeability generated dry *N. physalodes* fruits that irregularly bursts at the final stage of fruit maturation, releasing its numerous seeds into the environment (Horton, 1979; Ortiz-Ramírez et al., 2018).

The cutin deposition on *A. officinarum* fruits was substantially higher compared to *N. physalodes* fruits but similarly composed of almost exclusively 9/10,ω-dihydroxyhexadecanoic acid as basic cutin monomer. Cutin coverages of *Physalis* fruits were lower compared to *A. officinarum* fruits. Yet, their monomeric cutin composition was more complex and revealed a higher carbon chain length distribution containing 9,10-epoxy-ω-hydroxyoctadecanoic and 9,10,ω-trihydroxyoctadecanoic acids as main cutin monomers or, uncommonly, 9/10-oxo-ω-hydroxyhexadecanoic, 9/10,ω-dihydroxyoctadecanoic and 9,10,ω-trihydroxyoctadecanoic acids (Holloway, 1982). Hence, the cutin matrix of *Physalis, Alkekengi*, and *Nicandra* fruits was either quantitatively reduced and/or had a simplified monomeric composition.

Considering vast quantitative and compositional differences, the cutin matrix of ripe *Physalis* and *Alkekengi* fruits provided a supporting framework for cuticular waxes as an effective barrier against transpirational water loss. *Physalis* and *Alkekengi* species bore glossy and fleshy fruits such as *S. lycopersicum* and *C. annuum* that cross-link 9/10,ω-dihydroxyhexadecanoic acid as primary cutin monomer as commonly found in solanaceous fruits (Isaacson et al., 2009; Parsons et al., 2012; Yeats et al., 2012).

### Specifics of Solanoideae Inflated Fruiting Calyces

Inflated fruiting calyces are evolutionary adaptive features enveloping fruits, acting as a protective cover during their maturation. Micromorphologically, fruiting calyces of *Physalis, Alkekengi*, and *Nicandra* species shared characteristics and had few commonalities with their corresponding leaves. Predominantly, glandular and non-glandular trichomes were species-specifically distributed on the abaxial surface of hypostomatous *Physalis, Alkekengi*, and *Nicandra* fruiting calyces. This preferential distribution highlights the defensive role of the trichomes in the interaction between the fruiting calyx and the surrounding environment since they primarily covered the surface directly exposed to herbivore attacks and abiotic stresses (Dalin et al., 2008; Kariñho-Betancourt et al., 2015).

Initially green, fruiting calyces blended in with leaves, and the occurrence of stomata substantiated their photosynthetic activity. Entirely enclosing the maturing fruit, the fruiting calyx provided an internal microclimate that benefits fruit and seed development by contributing to a dynamic equilibrium between air humidity, thermoregulation, and light reflectance holding off rainfall and wind (Wilf et al., 2017; Li et al., 2019). Cuticular waxes on fruiting calyces were dominated by *n*-alkanes, *iso*- and *anteiso*-branched alkanes, *n*-alkanols, and alkanoic acids. The cuticular wax load of *P. ixocarpa, A. officinarum*, and *N. physalodes* fruiting calyces was even higher and more complex assembled than their corresponding leaves. Nevertheless, this protective envelope did not significantly reduce the water permeability of their enclosed fruits.

Exceptionally, *P. peruviana* fruiting calyces, with a small but *n*-alkane- and *n*-alkanol-rich cuticular wax coverage, most likely provided a low-transpirational demand environment that reduced the relatively high cuticular water permeability of their fruits during maturation, as similarly shown for *Physalis floridana* (Li et al., 2019). Accordingly, Fischer (1995) measured temperatures about 5°C lower inside this structure in comparison to the surrounding atmosphere, and it was also shown that *Physalis* enclosed fruits have lower weight loss and ethylene production and longer storage life than fruits without fruiting calyces (Fischer, 1995; Novoa et al., 2006; Balaguera-López et al., 2014). The results herein found for *P. peruviana* suggest that its fruiting calyx has a mechanism similar to the shown by *P. floridana* since the presence of this post-floral structure significantly affected the cuticular water loss of *P. peruviana* fruits.

Post-floral morphogenesis of *Physalis, Alkekengi*, and *Nicandra* fruiting calyces comprised changes in color, architecture, and extent. Several modifications proceeded simultaneously with fruit maturation but a correlation between fruit extent and enlargement of fruiting calyces did not exist. As soon as fruits ripened, fruiting calyces of *P. ixocarpa*, almost filled by their fruits of similar surface area, burst gradually. The red fruiting calyces of *A. officinarum* degraded with time, and the brightly colored, glossy, fleshy *Alkekengi* fruits became finally visible and attractive to frugivore seed dispersers. In contrast, *P. peruviana* and *N. physalodes* showed persistent fruiting calyces, but they dried up simultaneously with their encapsulated fruits in the latter. The highly fragile *N. physalodes* fruit released numerous seeds into the fruiting calyx that was unique for having auriculate calyx lobes that opened close to the base. Placed at a higher plant position, the deeply lobed *N. physalodes* fruiting calyx supported seed dispersal selectively by the wind. Fruiting calyces play an important role as a defense mechanism during fruit development and maturation, protecting their enclosed fruits against abiotic and biotic stresses (Herrera, 2005; Fischer et al., 2011; Li et al., 2019). Detached from the stem, inflated fruiting calyces have also been proposed to facilitate seed dispersal by rolling fruits through the wind as tumbleweeds or giving fruit floatation ability (Horton, 1979; Wilf et al., 2017; Li et al., 2019).

Having a more efficient transpiration barrier is crucial for avoiding fruit dehydration, which is a determining factor for the pre- and post-harvest quality and economic value of fruit crops (Lara et al., 2014; Riederer et al., 2015). Despite no influence of the fruiting calyx on water permeability observed for *P. ixocarpa* and *A. officinarum*, fruits of these two species had the most efficient cuticular transpiration barriers among plant organs and species investigated, whereas the fruiting calyx of *N. physalodes* might actually contribute to the fruits drying process. Moreover, our results suggest that, in addition to the role of the fruit cuticle in the minimization of water loss and modulation of fruit quality (Lara et al., 2014, 2015), the fruiting calyx protection, with its own cuticular properties, can further enhance fruit cuticle performance of the tropical *P. peruviana* fruits.

## Conclusion

To the authors' best knowledge, this is an unprecedented description of *P. peruviana, P. ixocarpa, A. officinarum*, and *N. physalodes* plant cuticle properties. Accordingly, this is the first comparative study focused on better understanding the cuticular structure, chemical, and functional composition of vegetative and reproductive plant organs from plant species bearing the inflated fruiting calyx as a special morphological trait. Placing this study in a developmental context, our results revealed species-specific surfaces of fruiting calyces, which post-floral modifications have the first impact on the maturation and diversity of *Physalis, Alkekengi*, and *Nicandra* fruits into fleshy or dry fruits and, finally, on their seed dispersal strategies. The cuticular barrier forms a continuous layer on the fruiting calyx surface, which is comparable to leaves, providing abiotic stress tolerance and improving biotic stress resistance of the fruiting calyx itself and the enclosed fruit. Accordingly, distinctly less effort was required for fruit cuticular wax and cutin deposition, for example, accumulation of pentacyclic triterpenoids. Overall, we were able to show commonalities in the cuticle composition, surface structure, and water permeability of leaves, inflated fruiting calyx, and fruits within the Solanoideae subfamily. Our results contribute to deepening our understanding of plant cuticle-associated traits and improvement of transpiration efficiency in plant species of agricultural and ornamental relevance.

## Data Availability Statement

The raw data supporting the conclusions of this article will be made available by the authors, without undue reservation.

## Author Contributions

AS, JL, and MR conceived the ideas and designed the methodology. AS and JL performed the experiments and analyzed the data. JL led the writing of the manuscript. AS led the revision of the manuscript. All authors contributed critically to the drafts and gave final approval for publication.

## Funding

AS was a recipient of a doctoral fellowship from the Brazilian National Council for Scientific and Technological Development (CNPq, grant number 290145/2015-1). This publication was supported by the Open Access Publication Fund of the University of Würzburg.

## Conflict of Interest

AS is currently affiliated with the company Syngenta Crop Protection. AS was not affiliated with Syngenta Crop Protection at the time of the study. The remaining authors declare that the research was conducted in the absence of any commercial or financial relationships that could be construed as a potential conflict of interest.

## Publisher's Note

All claims expressed in this article are solely those of the authors and do not necessarily represent those of their affiliated organizations, or those of the publisher, the editors and the reviewers. Any product that may be evaluated in this article, or claim that may be made by its manufacturer, is not guaranteed or endorsed by the publisher.

## References

[B1] Ahmad K. J. (1964). Cuticular studies in Solanaceae. Canadian Journal of Botany 42, 793–803. 10.1139/b64-071

[B2] Balaguera-López H. E. Martínez C. Andrea C. Herrera-Arévalo A. (2014). The role of the calyx in the postharvest behavior of cape gooseberry (*Physalis peruviana* L.) fruits ecotype Colombia. Revista Colombiana de Ciencias Hortícolas 8, 181–191. 10.17584/rcch.2014v8i2.3212

[B3] Bonaventure G. Beisson F. Ohlrogge J. Pollard M. (2004). Analysis of the aliphatic monomer composition of polyesters associated with *Arabidopsis* epidermis: occurrence of octadeca-*cis*-6, *cis*-9-diene-1, 18-dioate as the major component. Plant J. 40, 920–930. 10.1111/j.1365-313X.2004.02258.x 15584957

[B4] Bueno A. Alfarhan A. Arand K. Burghardt M. Deininger A. Hedrich R.. (2019). Effects of temperature on the cuticular transpiration barrier of two desert plants with water-spender and water-saver strategies. J. Exp. Bot. 70, 1613–1625. 10.1093/jxb/erz018 30715440 PMC6416792

[B5] da Luz B. R. (2006). Attenuated total reflectance spectroscopy of plant leaves: a tool for ecological and botanical studies. New Phytol. 172, 305–318. 10.1111/j.1469-8137.2006.01823.x 16995918

[B6] Dalin P. Ågren J. Björkman C. Huttunen P. Kärkkäinen K. (2008). “Leaf trichome formation and plant resistance to herbivory,” in Induced Plant Resistance to Herbivory, ed Andreas, S. (Dordrecht: Springer), 89–105. 10.1007/978-1-4020-8182-8_4

[B7] Deanna R. Barboza G. E. Carrizo García C. (2017). Phylogenetic relationships of Deprea: new insights into the evolutionary history of physaloid groups. Mol. Phylogenet. Evol. 119, 71–80. 10.1016/j.ympev.2017.11.001 29108936

[B8] Deanna R. Larter M. D. Barboza E. G. Smith S. D. (2019). Repeated evolution of a morphological novelty: a phylogenetic analysis of the inflated fruiting calyx in the Physalideae tribe (Solanaceae). Am. J. Bot. 106, 270–279. 10.1002/ajb2.1242 30779447

[B9] Diarte C. de Souza A. X. Staiger S. Deininger A.-C. Bueno A. Burghardt M.. (2021). Compositional, structural and functional cuticle analysis of *Prunus laurocerasus* L. sheds light on cuticular barrier plasticity. Plant Physiol. Biochem. 158, 434–445. 10.1016/j.plaphy.2020.11.028 33257229

[B10] Domínguez E. Heredia-Guerrero J. A. Heredia A. (2017). The plant cuticle: old challenges, new perspectives. J. Exp. Bot. 68, 5251–5255. 10.1093/jxb/erx389 29136457 PMC5853762

[B11] Dyki B. Jankiewicz L. S. Staniaszek M. (1997). Anatomy and surface micromorphology of tomatillo fruit (*Physalis ixocrpa* Brot.). Acta Soc. Bot. Pol. 66, 21–27. 10.5586/asbp.1997.003

[B12] Dyki B. Jankiewicz L. S. Staniaszek M. (1998). Anatomical structure and surface micromorphology of tomatillo leaf and flower (*Physalis ixocarpa* Brot., Solanaceae). Acta Soc. Bot. Pol. 67, 181–191. 10.5586/asbp.1998.021

[B13] Fernández V. Guzmán-Delgado P. Graça J. Santos S. Gil L. (2016). Cuticle structure in relation to chemical composition: re-assessing the prevailing model. Front. Plant Sci. 7, 427. 10.3389/fpls.2016.00427 27066059 PMC4814898

[B14] Fich E. A. Segerson N. A. Rose J. K. C. (2016). The plant polyester cutin: biosynthesis, structure, and biological roles. Annu. Rev. Plant Biol. 67, 207–233. 10.1146/annurev-arplant-043015-111929 26865339

[B15] Fischer G. (1995). QEffect of root zone temperature and tropical altitude on the growth, development and fruit quality of cape gooseberry (*Physalis peruviana* L.) (Doctoral Thesis). Humboldt University, Berlin, 171.

[B16] Fischer G. Herrera A. Almanza P. J. (2011). “Cape gooseberry (*Physalis peruviana* L.),” in Postharvest Biology and Technology of Tropical and Subtropical Fruits, eds Elhadi M. Y. (Sawston: Woodhead Publishing), 374–397. 10.1533/9780857092762.374

[B17] Grncarevic M. Radler F. (1967). The effect of wax components on cuticular transpiration – model experiments. Planta 75, 23–27. 10.1007/BF00380835 24550011

[B18] Guo Y. Jetter J. (2017). Comparative analyses of cuticular waxes on various organs of potato (*Solanum tuberosum* L.). J. Agric. Food Chem. 65, 3926–3933. 10.1021/acs.jafc.7b00818 28467851

[B19] Haliński Ł. P. Samuels J. Stepnowski P. (2017). Multivariate analysis as a key tool in chemotaxonomy of brinjal eggplant, African eggplants and wild related species. Phytochemistry 144, 87–97. 10.1016/j.phytochem.2017.09.001 28910606

[B20] He C. Münster T. Saedler H. (2004). On the origin of floral morphological novelties. FEBS Lett. 567, 147–151. 10.1016/j.febslet.2004.02.090 15165908

[B21] He C. Saedler H. (2005). Heterotopic expression of MPF2 is the key to the evolution of the Chinese lantern of *Physalis*, a morphological novelty in Solanaceae. Proc. Natl. Acad. Sci. U. S. A. 102, 5779–5784. 10.1073/pnas.0501877102 15824316 PMC556287

[B22] He C. Saedler H. (2007). Hormonal control of the inflated calyx syndrome, a morphological novelty, in *Physalis*. Plant J. 49, 935–946. 10.1111/j.1365-313X.2006.03008.x 17316177

[B23] Herrera C. M. (2005). Post-floral perianth functionality: contribution of persistent sepals to seed development in Helleborus foetidus (Ranunculaceae). Am. J. Bot. 92, 1486–1491. 10.3732/ajb.92.9.1486 21646166

[B24] Holloway P. J. (1982). “The chemical constitution of plant cutins,” in The Plant Cuticle, eds Cutler, D. F., Alvin, K. L., and Price, C. E. (London: Academic Press), 45–85.

[B25] Horton P. (1979). Taxonomic account of *Nicandra* (Solanaceae) in Australia. J. Adelaide Bot. Gard. 1, 351–356.

[B26] Hu J.-Y. Saedler H. (2007). Evolution of the inflated calyx syndrome in Solanaceae. Mol. Biol. Evol. 24, 2443–2453. 10.1093/molbev/msm177 17827172

[B27] Huang H. Burghardt M. Schuster A. C. Leide J. Lara I. Riederer M. (2017). Chemical composition and water permeability of fruit and leaf cuticles of *Olea europaea* L. J. Agric. Food Chem. 65, 8790–8797. 10.1021/acs.jafc.7b03049 28880084

[B28] Huot O. B. Nachappa P. Tamborindeguy C. (2013). The evolutionary strategies of plant defenses have a dynamic impact on the adaptations and interactions of vectors and pathogens. Insect Sci. 20, 297–306. 10.1111/1744-7917.12010 23955882

[B29] Isaacson T. Kosma D. K. Matas A. J. Buda G. J. He Y. Yu B.. (2009). Cutin deficiency in the tomato fruit cuticle consistently affects resistance to microbial infection and biomechanical properties, but not transpirational water loss. Plant J. 60, 363–377. 10.1111/j.1365-313X.2009.03969.x 19594708

[B30] Jetter R. Kunst L. Samuels A. L. (2006). “Composition of plant cuticular waxes,” in Biology of the Plant Cuticle, Vol. 23, eds Riederer, M., and Müller, C. (Oxford: Blackwell), 145–181.

[B31] Jetter R. Riederer M. (2016). Localization of the transpiration barrier in the epi- and intracuticular waxes of eight plant species: water transport resistances are associated with fatty acyl rather than alicyclic components. Plant Physiol. 170, 921–934. 10.1104/pp.15.01699 26644508 PMC4734581

[B32] Kariñho-Betancourt E. Agrawal A. A. Halitschke R. Núñez-Farfán J. (2015). Phylogenetic correlations among chemical and physical plant defenses change with ontogeny. New Phytol. 206, 796–806. 10.1111/nph.13300 25652325

[B33] Khanal B. P. Knoche M. (2017). Mechanical properties of cuticles and their primary determinants. J. Exp. Bot. 68, 5351–5367. 10.1093/jxb/erx265 28992090

[B34] Knapp S. (2002). Tobacco to tomatoes: a phylogenetic perspective on fruit diversity in the Solanaceae. J. Exp. Bot. 53, 2001–2022. 10.1093/jxb/erf068 12324525

[B35] Koch K. Bhushan B. Barthlott W. (2008). Diversity of structure, morphology and wetting of plant surfaces. Soft Matter 4, 1943–1963. 10.1039/b804854a

[B36] Kosma D. K. Nemacheck J. A. Jenks M. A. Williams C. E. (2010). Changes in properties of wheat leaf cuticle during interactions with Hessian fly. Plant J. 63, 31–43. 10.1111/j.1365-313X.2010.04229.x 20409001

[B37] Lara I. Belge B. Goulao L. F. (2014). The fruit cuticle as a modulator of postharvest quality. Postharvest Biol. Technol. 87, 103–112. 10.1016/j.postharvbio.2013.08.012

[B38] Lara I. Belge B. Goulao L. F. (2015). A focus on the biosynthesis and composition of cuticle in fruits. J. Agric. Food Chem. 63, 4005–4019. 10.1021/acs.jafc.5b00013 25850334

[B39] Leide J. Hildebrandt U. Reussing K. Riederer M. Vogg G. (2007). The developmental pattern of tomato fruit wax accumulation and its impact on cuticular transpiration barrier properties: effects of a deficiency in a β-ketoacyl-coenzyme A synthase (LeCER6). Plant Physiol. 144, 1667–1679. 10.1104/pp.107.099481 17468214 PMC1914139

[B40] Leide J. Nierop K. G. J. Deininger A.-C. Staiger S. Riederer M. de Leeuw J. W. (2020). Leaf cuticle analyses: implications for the existence of cutan/non-ester cutin and its biosynthetic origin. Ann. Bot. 126, 141–162. 10.1093/aob/mcaa056 32222770 PMC7304474

[B41] Li J. Song C. He C. (2019). Chinese lantern in *Physalis* is an advantageous morphological novelty and improves plant fitness. Sci. Rep. 9, 596. 10.1038/s41598-018-36436-7 30679462 PMC6345875

[B42] López-Casado G. Matas A. J. Domínguez E. Cuartero J. Heredia A. (2007). Biomechanics of isolated tomato (*Solanum lycopersicum* L.) fruit cuticles: the role of the cutin matrix and polysaccharides. J. Exp. Bot. 58, 3875–3883. 10.1093/jxb/erm233 17975209

[B43] Lucatti A. F. van Heusden A. W. de Vos R. C. H. Visser R. G. F. Vosman B. (2013). Differences in insect resistance between tomato species endemic to the Galapagos Islands. BMC Ecol. Evol. 13, 175. 10.1186/1471-2148-13-175 23972016 PMC3765935

[B44] Macel M. Visschers I. G. S. Peters J. L. van Dam N. M. de Graaf R. M. (2020). High concentrations of very long chain leaf wax alkanes of thrips susceptible pepper accessions (*Capsicum* spp). J. Chem. Ecol. 46, 1082–1089. 10.1007/s10886-020-01226-x 33089351 PMC7677282

[B45] Martin L. B. B. Rose J. K. C. (2014). There's more than one way to skin a fruit: formation and functions of fruit cuticles. J. Exp. Bot. 65, 4639–4651. 10.1093/jxb/eru301 25028557

[B46] Matzke K. Riederer M. (1991). A comparative study into the chemical constitution of cutins and suberins from *Picea abies* (L.) Karst., *Quercus robur* L., and *Fagus sylvatica* L. Planta 185, 233–245. 10.1007/BF00194066 24186347

[B47] McDowell E. T. Kapteyn J. Schmidt A. Li C. Kang J.-H. Descour A.. (2011). Comparative functional genomic analysis of *Solanum* glandular trichome types. Plant Physiol. 155, 524–539. 10.1104/pp.110.167114 21098679 PMC3075747

[B48] Medina-Medrano J. R. Almaraz-Abarca N. González-Elizondo M. S. Uribe-Soto J. N. González-Valdez L. S. Herrera-Arrieta Y. (2015). Phenolic constituents and antioxidant properties of five wild species of *Physalis* (Solanaceae). Bot. Stud. 56, 1–13. 10.1186/s40529-015-0101-y 28510833 PMC5430310

[B49] Moses T. Pollier J. Shen Q Soetaert S. Reed J. Erffelinck M.-L.. (2015). OSC2 and CYP716A14v2 catalyze the biosynthesis of triterpenoids for the cuticle of aerial organs of *Artemisia annua*. Plant Cell 27, 286–301. 10.1105/tpc.114.134486 25576188 PMC4330586

[B50] Nobel P. S. (2009). “Leaves and fluxes,” in Physicochemical And Environmental Plant Physiology, ed Nobel P. S .(Academic Press), 365–438.

[B51] Novoa R. H. Bojacá M. Galvis J. A. Fischer G. (2006). La madurez del fruto y el secado del cáliz influyen en el comportamiento poscosecha de la uchuva, almacenada a 12 C (*Physalis peruviana* L.). Agron. Colomb. 24, 77–86.

[B52] Ortiz-Ramírez C. I. Plata-Arboleda S. Pabón-Mora N. (2018). Evolution of genes associated with gynoecium patterning and fruit development in Solanaceae. Ann. Bot. 121, 1211–1230. 10.1093/aob/mcy007 29471367 PMC5946927

[B53] Pabón-Mora N. Litt A. (2011). Comparative anatomical and developmental analysis of dry and fleshy fruits of Solanaceae. Am. J. Bot. 98, 1415–1436. 10.3732/ajb.1100097 21875970

[B54] Parsons E. P. Popopvsky S. Lohrey G. T. Lü S. Alkalai-Tuvia S. Perzelan Y.. (2012). Fruit cuticle lipid composition and fruit post-harvest water loss in an advanced backcross generation of pepper (*Capsicum* sp.). Physiol. Plant. 146, 15–25. 10.1111/j.1399-3054.2012.01592.x 22309400

[B55] Petit J. Bres C. Just D. Garcia V. Mauxion J.-P.-Marion D. Bakan B.. (2014). Analyses of tomato fruit brightness mutants uncover both cutin-deficient and cutin-abundant mutants and a new hypomorphic allele of GDSL lipase. Plant Physiol. 164, 888–906. 10.1104/pp.113.232645 24357602 PMC3912114

[B56] Petit J. Bres C. Mauxion J. P. Bakan B. Rothan C. (2017). Breeding for cuticle-associated traits in crop species: traits, targets, and strategies. J. Exp. Bot. 68, 5369–5387. 10.1093/jxb/erx341 29036305

[B57] Riederer M. Arand K. Burghardt M. Huang H. Riedel M. Schuster A. C.. (2015). Water loss from litchi (*Litchi chinensis*) and longan (*Dimocarpus longan*) fruits is biphasic and controlled by a complex pericarpal transpiration barrier. Planta 242, 1207–1219. 10.1007/s00425-015-2360-y 26159434

[B58] Riederer M. Schreiber L. (2001). Protecting against water loss: analysis of the barrier properties of plant cuticles. J. Exp. Bot. 52, 2023–2032. 10.1093/jexbot/52.363.2023 11559738

[B59] Rodríguez-López M. J. Garzo E. Bonani J. P. Fereres A. Fernández-Muñoz R. Moriones E. (2011). Whitefly resistance traits derived from the wild tomato *Solanum pimpinellifolium* affect the preference and feeding behavior of *Bemisia tabaci* and reduce the spread of Tomato yellow leaf curl virus. Phytopathology 101, 1191–1201. 10.1094/PHYTO-01-11-0028 21615206

[B60] Samuels L. Kunst L. Jetter R. (2008). Sealing plant surfaces: cuticular wax formation by epidermal cells. Ann. Rev. Plant Biol. 59, 683–707. 10.1146/annurev.arplant.59.103006.093219 18251711

[B61] Schreiber L. (2010). Transport barriers made of cutin, suberin and associated waxes. Trends Plant Sci. 15, 546–553. 10.1016/j.tplants.2010.06.004 20655799

[B62] Schreiber L. Riederer M. (1996). Ecophysiology of cuticular transpiration: comparative investigation of cuticular water permeability of plant species from different habitats. Oecologia 107, 426–432. 10.1007/BF00333931 28307383

[B63] Schuster A.-C. Burghardt M. Riederer M. (2017). The ecophysiology of leaf cuticular transpiration: are cuticular water permeabilities adapted to ecological conditions? J. Exp. Bot. 68, 5271–5279. 10.1093/jxb/erx321 29036342

[B64] Silva K. N. Agra M. F. (2005). Estudo farmacobotânico comparativo entre *Nicandra physalodes* e *Physalis angulata* (Solanaceae). Rev. Bras. Farmacogn. 15, 344–351. 10.1590/s0102-695x2005000400016

[B65] Sukhorukov A. P. (2008). Fruit anatomy of the genus *Anabasis* (Salsoloideae, Chenopodiaceae). Aust. Syst. Bot. 21, 431–442. 10.1071/sb08013

[B66] Sukhorukov A. P. (2012). Taxonomic notes on *Dysphania* and *Atriplex* (Chenopodiaceae). Willdenowia 42, 169–180. 10.3372/wi.42.42202

[B67] Szakiel A. Paczkowski C. Pensec F. Bertsch C. (2012). Fruit cuticular waxes as a source of biologically active triterpenoids. Phytochem. Rev. 11, 263–284. 10.1007/s11101-012-9241-9 23519009 PMC3601259

[B68] Tsubaki S. Sugimura K. Teramoto Y. Yonemori K. Azuma J. I. (2013). Cuticular membrane of Fuyu persimmon fruit is strengthened by triterpenoid nano-fillers. PLoS ONE 8, e75275. 10.1371/journal.pone.0075275 24086493 PMC3782500

[B69] Vogg G. Fischer S. Leide J. Emmanuel E. Jetter R. Levy A. A.. (2004). Tomato fruit cuticular waxes and their effects on transpiration barrier properties: functional characterization of a mutant deficient in a very-long-chain fatty acid β-ketoacyl-CoA synthase. J. Exp. Bot. 55, 1401–1410. 10.1093/jxb/erh149 15133057

[B70] Wilf P. Carvalho M. R. Gandolfo M. A. Cúneo N. R. (2017). Eocene lantern fruits from Gondwanan Patagonia and the early origins of Solanaceae. Science 355, 71–75. 10.1126/science.aag2737 28059765

[B71] Xu X. Feng J. Lü S. Lohrey G. T. An H. Zhou Y.. (2014). Leaf cuticular lipids on the Shandong and Yukon ecotypes of saltwater cress, *Eutrema salsugineum*, and their response to water deficiency and impact on cuticle permeability. Physiol. Plant. 151, 446–458. 10.1111/ppl.12127 24215503

[B72] Yeats T. H. Buda G. J. Wang Z. Chehanovsky N. Moyle L. C. Jetter R.. (2012). The fruit cuticles of wild tomato species exhibit architectural and chemical diversity, providing a new model for studying the evolution of cuticle function. Plant J. 69, 655–666. 10.1111/j.1365-313X.2011.04820.x 22007785 PMC3736592

[B73] Yeats T. H. Rose J. K. C. (2013). The formation and function of plant cuticles. Plant Physiol. 163, 5–20. 10.1104/pp.113.222737 23893170 PMC3762664

[B74] Zamora-Tavares M. P. Martínez M. Magallón S. Guzmán-Dávalos L. Vargas-Ponce O. (2016). *Physalis* and physaloids: a recent and complex evolutionary history. Mol. Phylogenet. Evol. 100, 41–50. 10.1016/j.ympev.2016.03.032 27063196

