# Supplementary material for: Multifunctional Contribution of the Inflated Fruiting Calyx: Implication for Cuticular Barrier Profiles of the Solanaceous Genera Physalis, Alkekengi, and Nicandra
Source: Front Plant Sci. 2022 Jul 6;13:888930. doi: 10.3389/fpls.2022.888930 (PMC9298275; doi:10.3389/fpls.2022.888930)
Supplement: Supplementary file 1 [file Presentation_1.pdf]

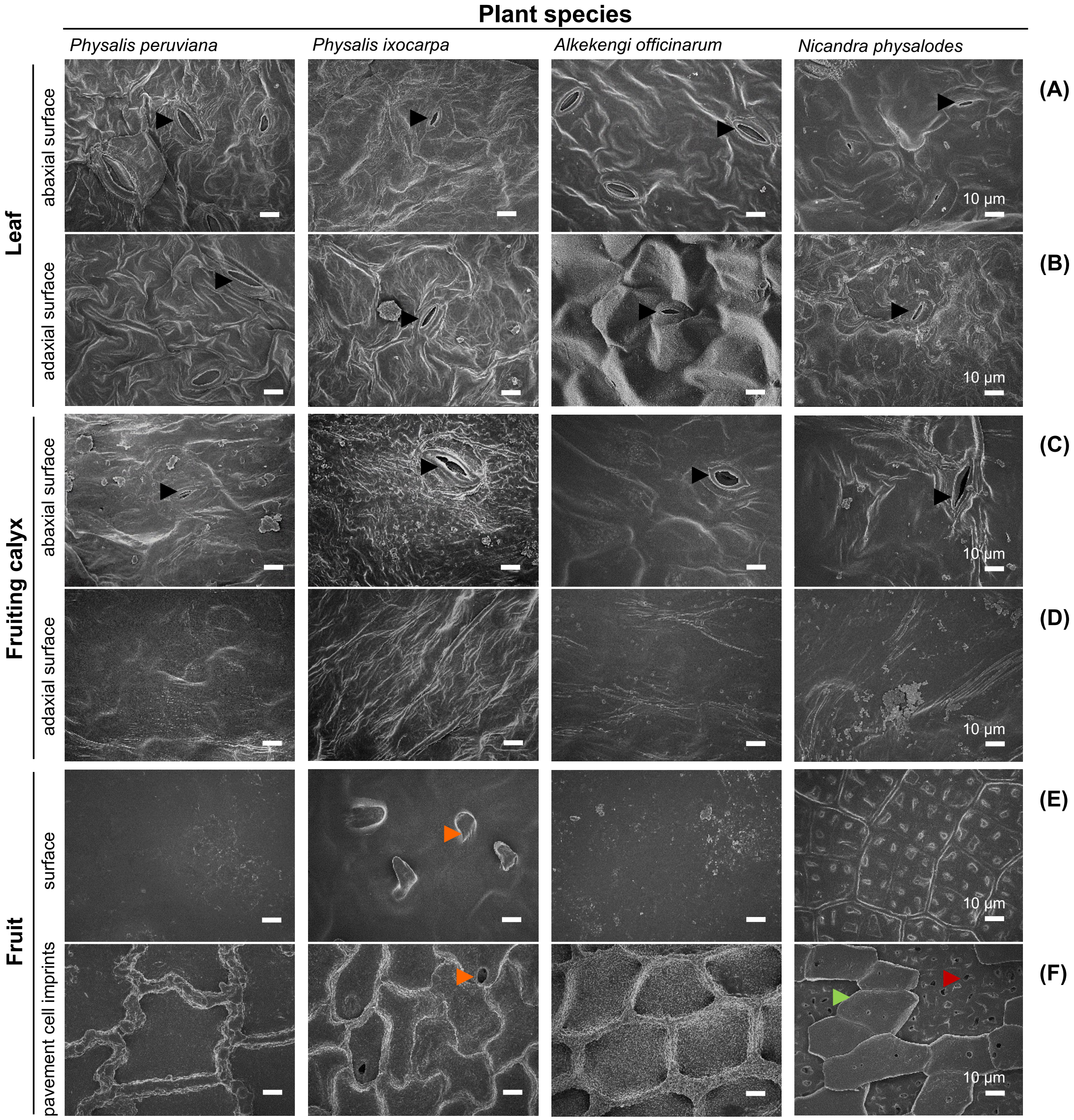

Figure S1 Micromorphological characteristics of the leaf (a, b), the fruiting calyx (c, d) and the fruit surface (e, f) of *Physalis, Alkekengi* and *Nicandra* species. Scanning electron microscopy was utilised for examining the abaxial and adaxial sides of fully expanded leaves and fully inflated fruiting calyces. Using enzymatically isolated fruit cuticles, the surface of ripe fruits and, on its reverse side, imprints of pavement cells were scanned. Arrowheads indicate stomata (black), cuticular protuberances/invaginations (dark orange), sclerified cells (green) and epidermal pores (red).

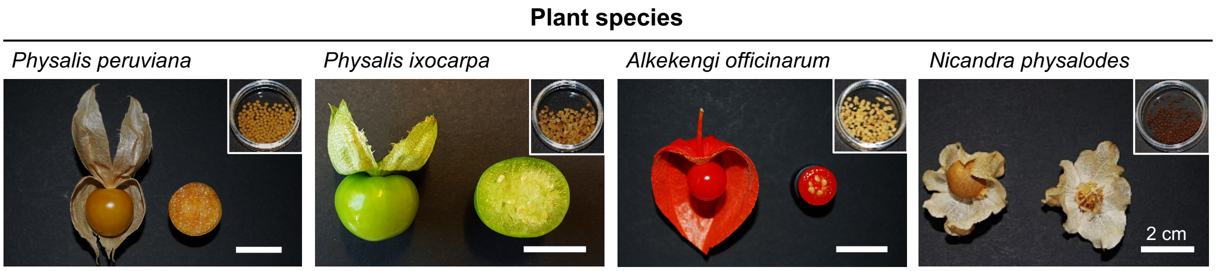

Figure S2 Morphological characteristics of fruiting calyces, fruits and seeds of *Physalis, Alkekengi* and *Nicandra* species.
